# Dedifferentiation Does Not Account for Hyperconnectivity after Traumatic Brain Injury

**DOI:** 10.3389/fneur.2017.00297

**Published:** 2017-07-17

**Authors:** Rachel Anne Bernier, Arnab Roy, Umesh Meyyappan Venkatesan, Emily C. Grossner, Einat K. Brenner, Frank Gerard Hillary

**Affiliations:** ^1^Department of Psychology, Pennsylvania State University, University Park, PA, United States; ^2^Social Life and Engineering Sciences Imaging Center, University Park, PA, United States; ^3^Department of Neurology, Hershey Medical Center, Hershey, PA, United States

**Keywords:** traumatic brain injury, TBI, functional connectivity, dedifferentiation, graph theory

## Abstract

**Objective:**

Changes in functional network connectivity following traumatic brain injury (TBI) have received increasing attention in recent neuroimaging literature. This study sought to understand how disrupted systems adapt to injury during resting and goal-directed brain states. Hyperconnectivity has been a common finding, and dedifferentiation (or loss of segregation of networks) is one possible explanation for this finding. We hypothesized that individuals with TBI would show dedifferentiation of networks (as noted in other clinical populations) and these effects would be associated with cognitive dysfunction.

**Methods:**

Graph theory was implemented to examine functional connectivity during periods of task and rest in 19 individuals with moderate/severe TBI and 14 healthy controls (HCs). Using a functional brain atlas derived from 83 functional imaging studies, graph theory was used to examine network dynamics and determine whether dedifferentiation accounts for changes in connectivity. Regions of interest were assigned to one of three groups: task-positive, default mode, or other networks. Relationships between these metrics were then compared with performance on neuropsychological tests.

**Results:**

Hyperconnectivity in TBI was most commonly observed as increased within-network connectivity. Network strengths within networks that showed differences between TBI and HCs were correlated with performance on five neuropsychological tests typically sensitive to deficits commonly reported in TBI. Hyperconnectivity within the default mode network (DMN) during task was associated with better performance on Digit Span Backward, a measure of working memory [*R*^2^(18) = 0.28, *p* = 0.02]. In other words, increased differentiation of networks during task was associated with better working memory. Hyperconnectivity within the task-positive network during rest was not associated with behavior. Negative correlation weights were not associated with behavior.

**Conclusion:**

The primary hypothesis that hyperconnectivity occurs through dedifferentiation was not supported. Instead, enhanced connectivity post injury was observed within network. Results suggest that the relationship between increased connectivity and cognitive functioning may be both state (rest or task) and network dependent. High-cost network hubs were identical for both rest and task, and cost was negatively associated with performance on measures of psychomotor speed and set-shifting.

## Introduction

In recent years, there has been a shift in the literature toward implementing neuroimaging techniques such as functional magnetic resonance imaging to study how the brain changes at a *systems* level after injury. This conceptual shift is based upon the growing interest over the past decade in examining the functional connectivity of the system of networks underlying brain functioning. Functional connectivity refers to the correlation between brain signals derived from distinct regions of interest (ROIs). One approach to examining large-scale network changes after traumatic brain injury (TBI) is through the use of graph theory, which is a branch of mathematics permitting the analysis of the connections between the members (nodes) of a network (1, 2). Recent focus in network neuroscience has been to understand the functioning of several large-scale networks, including the default mode network (DMN). The DMN has a number of regional contributions including the posterior cingulate cortex and medial prefrontal cortex (3). This group of brain structures appears to be most metabolically demanding when an individual is awake but not engaged in goal-oriented behavior (4, 5).

Detailed analysis of DMN functioning reveals that its components may play a number of roles in information processing (6), but most commonly it maintains a reciprocal relationship with the task-positive or other networks supporting goal-directed behavior (7, 8). The organization of the brain into networks is thought to reflect efficient organization and functioning (9, 10) such that metabolic cost is minimized while maximizing efficiency. Such segregation of neural networks may be linked to better cognitive functioning (11). For example, suppression of the DMN during goal-oriented behavior has been associated with better task performance in healthy adults (12), as well as in chronic TBI (6).

Finally, given that positive and negative connectivity may represent distinct neural processes (13, 14), to examine dedifferentiation, we separate positive and negative connectivity effects (i.e., positive and negative correlations). This was first observed in a small sample of subjects during the first 6 months post injury (15) and later cross-sectional work demonstrated similar effects. In a mixed sample of mild and moderate TBI, Sharp et al. (16) reported that hyperconnectivity within the DMN at rest predicted less cognitive impairment and faster reaction time. In our prior work examining working memory functioning, we demonstrated that connectivity increases followed task load and these effects were more pronounced post injury (17, 18). This finding of enhanced connectivity has been observed elsewhere, including both longitudinally during recovery (17–20) and in cross-sectional studies of chronic samples of TBI (21–24). In the study of Dobryakova et al. (22), increased interhemispheric connectivity was observed during a novel working memory task when comparing TBI and healthy control (HC) samples. In the current study, we focus on chronic TBI to determine if residual effects of hyperconnectivity can be accounted for by dedifferentiation in network functioning.

### Dedifferentiation as a Mechanism for Hyperconnectivity

Given the common finding of regional increases in functional connectivity post injury, we sought to understand how disrupted systems adapt to injury both during resting and goal-directed brain states, and whether dedifferentiation, or loss of segregation of intrinsic neural networks, is accounting for hyperconnectivity effects. The term dedifferentiation has been typically used to describe the loss of specialization in the response of brain networks and is now considered a hallmark feature of normal aging (25, 26). In aging, dedifferentiation has been particularly evident among areas such as the prefrontal cortex, hippocampus, and primary sensory cortices (27–30). Several models have been developed to explain the apparent loss of segregation of neural networks in aging, including the hemispheric asymmetry reduction in older adults model, based on the observation of decreased interhemispheric lateralization of the prefrontal cortex. Alternatively, the compensation-related utilization of neural circuits hypothesis model posits that the increased involvement of previously differentiated regions reflects the use of alternate patterns of connectivity to perform a task, and that dedifferentiation thus serves as a compensatory mechanism (31, 32).

In order to examine dedifferentiation as a mechanism for hyperconnectivity, we use task and rest to examine properties *within* networks (e.g., connectivity between nodes within an intrinsic network), as well as properties *between* networks (i.e., connectivity between nodes from different intrinsic networks). One consideration is that neurological injury results in “network randomization” (33), reducing differentiation of established brain networks. Such dedifferentiation, or reduced specificity in network response between the brain’s networks, could be a marker of response to neural disruption and recovery. Determining whether connectivity changes occur within a given network, between networks, or both between and within networks is critical in order to understand what these connectivity changes mean for cognition and function. Thus far, studies have used graph theory metrics to examine TBI within rest and task, respectively, and have demonstrated that changes in these metrics are associated with cognitive deficit. This study seeks to expand upon the current literature and implement graph theory metrics (e.g., network strength) to examine how the relationship between connectivity during rest and task differs between TBI and HCs, and how this relationship is associated with cognition.

To examine the relationship between dedifferentiation and hyperconnectivity observed in TBI, we use three approaches. First, in order to induce network segregation, we examine connectivity during periods of rest and task. Second, we examine connectivity changes both within and between networks (e.g., DMN and the task-positive network). Finally, given that dedifferentiation has implications for how networks relate to one another, we separate positive and negative connectivity effects (i.e., positive and negative correlations), as positive and negative networks have been shown to have different network characteristics from one another (14).

To test the hypotheses regarding dedifferentiation, we created brain networks for graph analysis using a functional brain atlas derived from 83 functional imaging studies (34). We specifically chose this parcellation approach for two reasons. First, it is defined based upon the results of the most common functional imaging studies and we are interested in examining alterations in functional connectivity and implications for behavior and recovery. Second, the Power 264 parcellation provides near whole-brain coverage with greater specificity than most anatomical atlases. In a recent study, this approach afforded greater opportunity to observe fractionation of networks in TBI compared to a driven approach (35). The goal was to first replicate findings in moderate and severe chronic TBI, where investigators have observed hyperconnectivity in core networks [e.g., DMN; for review see Ref. (36, 37)]. Second, we aimed to determine if hyperconnectivity could be explained by dedifferentiation of networks by comparing network response during task and rest, and if so, which nodes were driving these altered relationships (i.e., most costly nodes, or “hubs”). Finally, we sought to determine if the degree of within and between network connectivity was a predictor of cognitive functioning.

We hypothesized that individuals with TBI will show hyperconnectivity compared to HCs and that increased connectivity will be attributable to dedifferentiation of networks. Support for dedifferentiation would be found if the hyperconnectivity occurred through recruitment of ROIs from other networks with the DMN during rest and with recruitment of ROIs from other networks and the task-positive network during task. We also hypothesize that positive hyperconnectivity will be associated with decreased performance on measures sensitive to deficit in TBI. Finally, we predicted that less negative weighted network strength will be associated with worse performance on cognitive measures, based upon findings from the mild TBI (mTBI) literature (38, 39).

## Materials and Methods

### Procedure

This study was a retrospective analysis of functional imaging data and neuropsychological testing data previously collected. All participants underwent a similar MRI protocol, which included a resting state scan in addition to a block design one-back task, followed by administration of a traditional neuropsychological battery outside of the scanner. Imaging data were collected on a Philips Achieva 3T scanner or a Siemens Magnetom Trio 3T whole-body scanner housed in the Department of Radiology at Hershey Medical Center, or either a Siemens Magnetom Trio 3T whole-body scanner or a Siemens Prisma 3 T whole-body scanner, both housed in the Social, Life, and Engineering Sciences Imaging Center at The Pennsylvania State University, University Park. Informed consent was gathered for all participants. The Penn State IRB approved the informed consent form that was used in this study. Additionally, participants were compensated for participating in this study.

### Participants

Participants included 19 individuals with moderate to severe TBI, indicated by a Glasgow Coma Scale score of 3–12 (40) or by positive MRI or CT finding, who were at least approximately 1 year post injury. These individuals were recruited as part of one of two possible studies: a cross-sectional study at least 12 months post injury, and a longitudinal study examining recovery beginning 3 months post injury. Data from the longitudinal study used was collected from the third time point, approximately 12 months post injury. Though this method of recruitment yields a sample in which a portion of the participants have previously been exposed to the stimuli, tests that were administered were chosen because they have been shown to have little effect with practice (e.g., one-back task, Digit Span subtest from the Wechsler Adult Intelligence Scale-III). To examine this effect directly, an independent samples *t*-test comparing mean performance on all cognitive tests showed no group differences between individuals with TBI recruited from the cross-sectional study and those recruited from the longitudinal study who had been exposed to the stimuli before. Thus, it is unlikely that practice effects accounted for the results discussed below. A group of age- and education-matched HCs underwent the identical protocol and time 2 data for this sample were used in order to equilibrate exposure effects. See Table 1 for demographic information.

**Table 1:** Demographic information.

	Sample size *n*	Age; mean (SD)	Education; mean (SD)	Gender	Glasgow Coma Scale; mean (SD)	Time postinjury (years); mean (SD)
Traumatic brain injury	19	29.52 (13.05)	13.35 (2.21)	8 F, 11 M	7 (4.35)	1.88 (2.5)
Health control	14	40.07 (17.49)	13.29 (1.77)	4 F, 10 M	N/A	N/A

### Measures

#### MRI Scans

##### T1-Weighted Magnetization-Prepared Rapid Acquisition with Gradient Echo

Structural data were acquired with 1 mm × 1 mm × 1 mm voxels, a repetition time (TR) of 2,300 ms, and an echo time (TE) of 2.98 ms, and slices were collected interleaved.

##### Resting State Scan

All participants were presented with the same stimulus, were instructed to fixate on the white cross on the center of the screen, and were reminded not to fall asleep. The 34–35 slices collected were interleaved with 3 mm × 3 mm × 4 mm voxels and acquired with a TR of 2,000 ms and an TE of 30 ms. The first five volumes were removed prior to beginning preprocessing, leaving 145 volumes for analysis.

##### One-Back Task

This task is an established block-design task with low cognitive demand where individuals are presented with a series of letters one at a time. A button press with the right hand on the right grip is required if the presented letter matched the letter that preceded it or to press the button with their left hand on the left grip if the presented letter did not match the letter that preceded it. Scan parameters for the *n*-back task were identical to the resting state scan, with slices collected interleaved with 3 mm × 3 mm × 4 mm voxels, and have a TR of 2,000 ms and a TE of 30 ms. The first 10 volumes were discarded, resulting in 126 volumes included in analyses.

#### Neuropsychological Tests

In addition to completing the one-back task inside the scanner, all participants were administered a battery of neuropsychological tests outside of the scanner aimed at capturing areas of cognition known to be affected by TBI, such as working memory, processing speed, and executive functioning. The battery included Wechsler Adult Intelligence Test-III Digit Span Backward and Forward, Trail Making Test A and B, and Visual Search and Attention Task (VSAT).

### Data Analysis

#### Data Preprocessing

All individuals’ data underwent the same preprocessing pipeline. Each individual’s working memory and resting state data were slice-timed corrected, realigned, normalized to a standard T1 template from the Montreal Neurological Institute, and smoothed to minimize signal-to-noise ratio. Data were preprocessed using SPM8, and motion was examined using the Artifact Repair toolbox (41), in order to identify subjects with large motion and correct for perturbations in the BOLD signal due to motion. Individuals with greater than 25% volumes requiring interpolation due to motion during either rest or task were discarded from analyses, as recommended by Mazaika et al. (41). As a result, of the original 22 eligible subjects with TBI, one case was discarded due to motion during rest, one was discarded due to motion during task, and a third was excluded due to aberrant BOLD signal, yielding a sample of 19 subjects with TBI. Of the original sample of 15 HCs, one was discarded due to excessive motion during the rest scan.

#### ROI Selection

Power’s 264 functionally defined atlas was used to define ROIs (34). Whole brain connectivity was examined across these 264 ROIs using graph theoretical methods (explained below). Power and colleagues’ functional labels for each ROI were then used to group these ROIs into five functional networks (see Table 2). The primary goal of grouping these ROIs into networks was to be able to determine whether connectivity changes observed in individuals with TBI compared to HCs were occurring in networks typically functionally associated with other brain states (e.g., task-related network recruited during rest).

**Table 2:** Power regions of interest (ROI) networks.

Network	Number of ROIs	Examples of included ROIs
1. Task+	59	Frontoparietal task control
2. Default mode network	58	Default mode
3. Other networks	147	Others

#### Graph Theory

**Figure 1:**
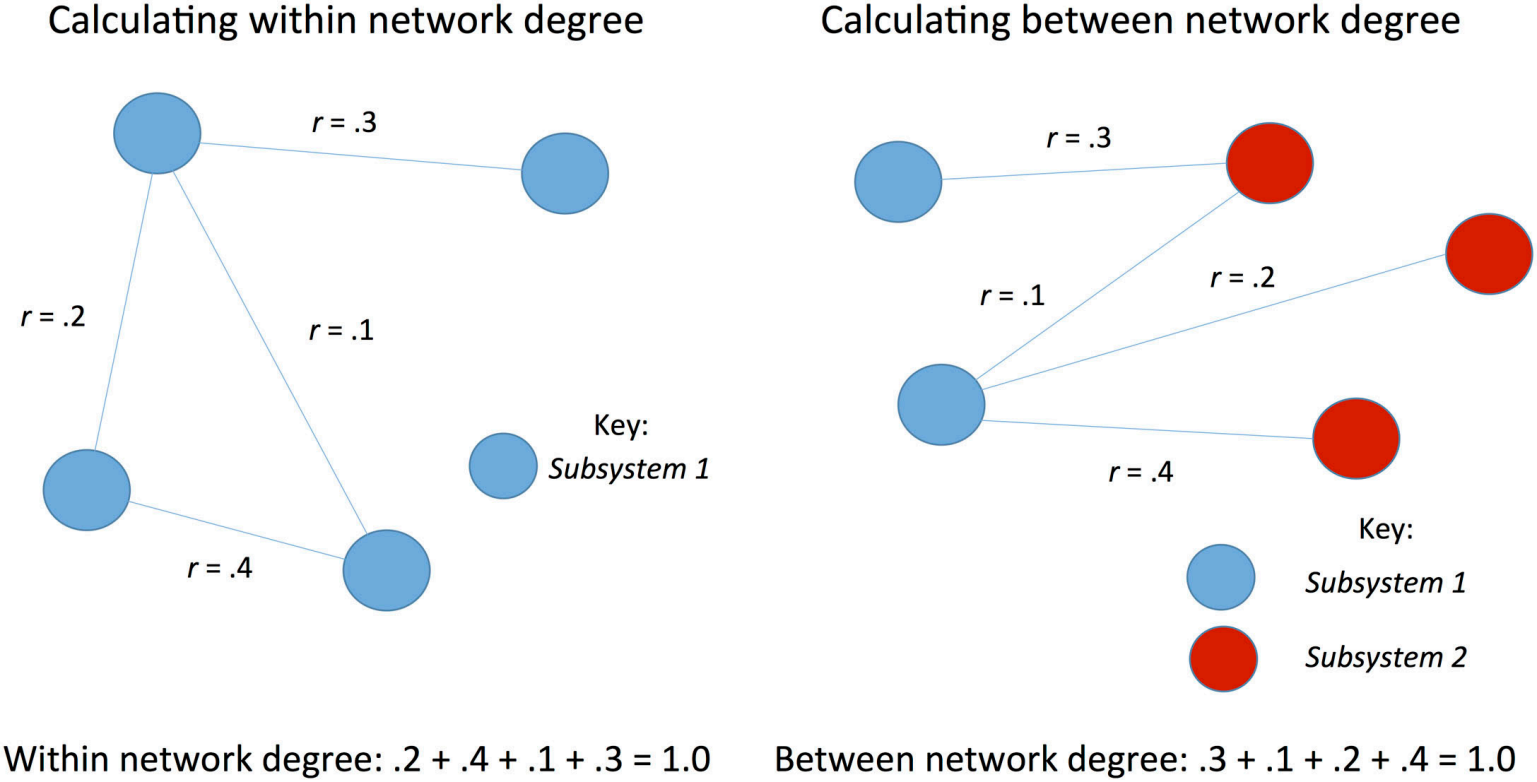
Schematic of calculation of network strength.

Graph theory was used to examine whole brain connectivity and determine patterns of response. Power ROIs (34) were correlated with each other to form an adjacency matrix using a code written in R [R.3.1.1 (42)] [e.g., *N* ROIs will produce N*(*N* − 1)/2 undirected connections]. Out of these N*(*N* − 1)/2 correlation values only those that survived FDR test at 0.05 were chosen and the remaining connections were set to 0. To test our hypothesis of dedifferentiation of networks, between and within network strength was calculated for each network during both rest and task for both positive and negative connectivity (see Figure 1). In order to assess whether functional connectivity differences were associated with better or worse recovery, several metrics, including network strength and degree of nodes (see Table 3), were correlated with cognitive functioning using SPSS (version 24.0).

**Table 3:** Graph theory terms.

Graph theory term	Definition	Computation
Edge	Significant correlation between time series of two different nodes (i.e., regions of interest)	

Network strength	Total number of edges by the weight of those edges (in weighted graph) can be examined globally or within a more specific network determined *a priori*	$S=\underset{j\in N\left(i\right)}{\sum }w(e_{ij})$

Degree	Absolute sum or total number of connections emanating from a particular node	${\text{deg}}_{i}=\sum_{j\in N\left(i\right)}w(e_{ij})$, where *N*(*i*) is the set of neighbors of node *i*.

Clustering coefficient (*C*)	The number of edges that exist between a node and its nearest neighbors	by $\bar{C}=\frac{1}{n}\sum_{i=1}^{n}C_{i}$, where $C_{i}=\frac{2\left|\left\{e_{jk}:j,k\in N\left(i\right),e_{jk}\in E\right\}\right|}{{\text{deg}}_{i}\left({\text{deg}}_{i}-1\right)}$

Path length (*L*)	The average minimum number of edges required to travel between two given nodes	As $\bar{L}=\frac{1}{n(n-1)}\sum_{i\neq j}d(i,j)$, where *d*(*i*,*j*) is the shortest distance between node *i* and node *j*

## Results

### Analysis of Whole-Brain Graph Metrics

Individuals with TBI and HCs did not differ significantly in terms of mean path length or mean clustering among significant positive and negative edges in the whole brain, respectively, during task or rest. Of note, mean clustering during rest was interpreted as higher in individuals with TBI (M = 0.023, SD = 0.006) than HCs (M = 0.02, SD = 0.005), Cohen’s *d* = 0.54, *p* = 0.069.

### Whole Brain Differences in Weighted Network Strength

**Figure 2:**
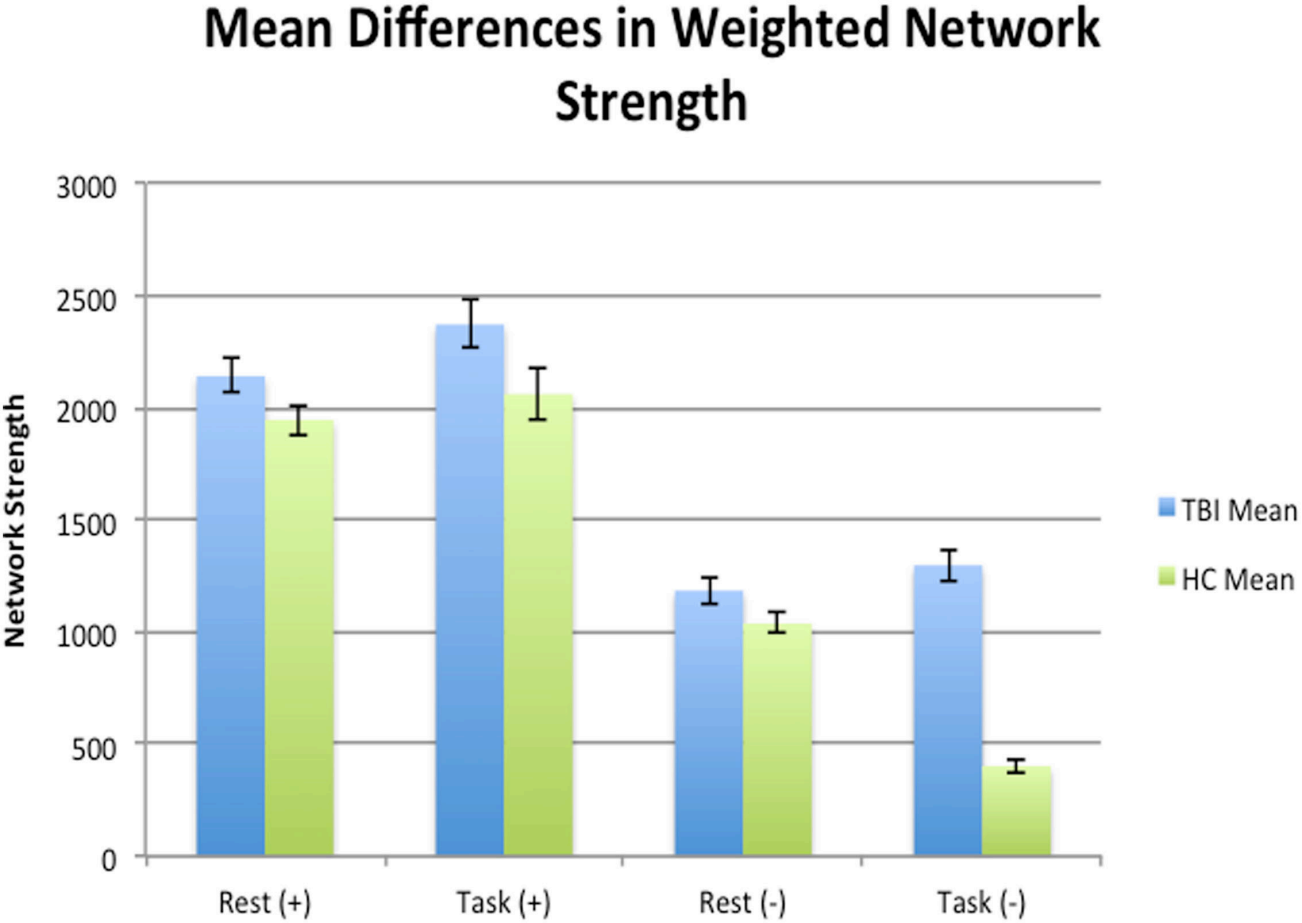
Mean differences at a global level in terms of mean network strength between health controls (HCs) and traumatic brain injury (TBI) during rest and task. Individuals with TBI were negatively hyperconnected at a global level compared to HCs during rest.

An independent samples *t*-test was used to test for global differences in mean network strength between individuals with TBI and HCs. During task mean negative network strength was significantly higher in individuals with TBI (M = 1,297.12, SD = 308.11) compared to HCs (M = 402.19, SD = 109.75) [*t*(31) = −11.69, Cohen’s *d* = 3.87, *p* < 0.001], whereas positive connectivity during task was only marginally higher in those with TBI (M = 2,370.24, SD = 464.54) than HCs (M = 2,059.44, SD = 447.41) [(*t*) = −1.940, Cohen’s *d* = 0.68, *p* = 0.062]. Global mean network strength during rest showed only marginal significance for positive connectivity between TBI (M = 2,139.24, SD = 332.06) and HCs (M = 1,944.74, SD = 251.40) [(*t*) = −1.835, Cohen’s *d* = 0.66, *p* = 0.065] and for negative connectivity between TBI (M = 1,183.84, SD = 258.53) and HCs (M = 1,036.80, SD = 167.53) [(*t*) = −1.979, Cohen’s *d* = 0.68, *p* = 0.057], see Figure 2.

Pairwise *t*-test analyses were used to test for mean differences in total network strength between states within each sample. Mean network strength of negative edges was significantly different in HCs, decreasing from 1,036.79 (SD = 167.53) during rest to 402.19 (SD = 109.75) during task [*t*(13) = 11.946, Cohen’s *d* = 4.48, *p* = < 0.001]. Positive network strength did not differ for HCs between states (M = 1,944.74, SD = 251.39 during rest; M = 2,059.44, SD = 447.41 during task, Cohen’s *d* = −0.31, *p* = 0.386). Individuals with TBI showed no significant difference in mean network strength in terms of positive edges during rest (M = 2,139.23, SD = 332.06) and task (M = 2,370.24, SD = 464.54) [*t*(18) = −1.720, Cohen’s *d* = −0.57, *p* = 0.103] and in terms of negative edges between rest (M = 1,183.83, SD = 258.54) and task (M = 1,297.12, SD = 308.11) [*t*(18) = −1.672, Cohen’s *d* = −0.39, *p* = 0.112]. In other words, HCs were more negatively connected during rest compared to task, whereas individuals with TBI showed no global differences between states for either positive or negative connection weights.

At a global level, individuals with TBI only show hyperconnectivity during task for negative connectivity, but not for positive connectivity. There were no global differences between TBI and HC and the global level during rest. However, the lack of global mean differences in connectivity during task at the global level could be consistent with our hypothesis that posits hyperconnectivity compared to HCs *via* dedifferentiation of networks; in other words, differences in networks at the local level may be washing out effects at the global level.

### Subnetwork Differences in Weighted Network Strength

**Figure 3:**
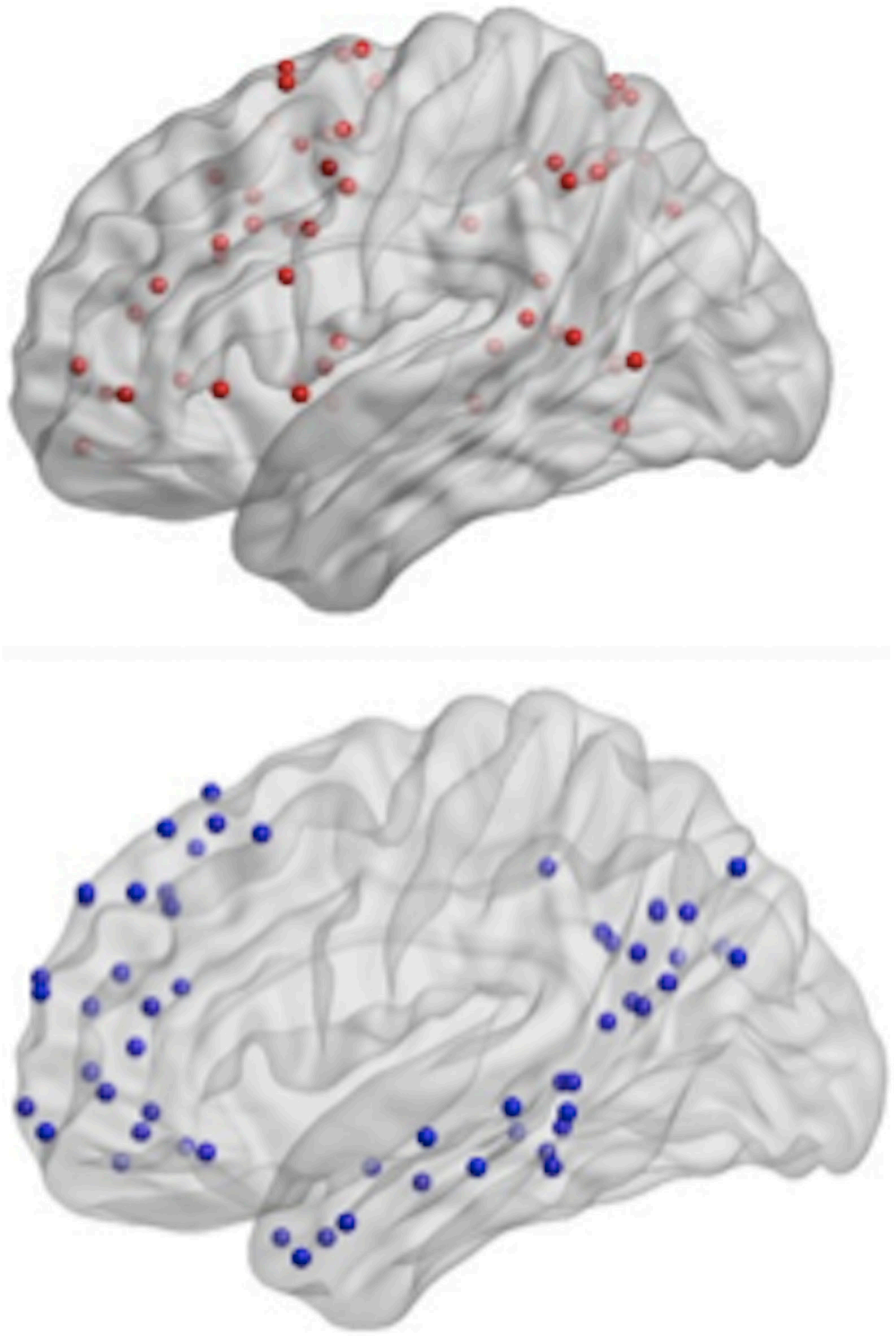
Top-power regions of interest (ROIs) recoded as the task-related network; bottom- Power ROIs recoded as the default mode network.

In order to test a dedifferentiation hypothesis, Power’s 264 ROIs (34) were assigned to one of three networks: task-related, default mode, and other networks (see Figure 3, Table 2). Analyses focused on mean weighted network strength between ROIs *within* the task-related network and within the default mode, respectively, in addition to *internetwork* mean weighted network strength involving the task network (i.e., valid links between task ROIs and ROIs from other networks) and the default mode (i.e., valid links between DMN ROIs and ROIs from other networks).

To examine the relationship between HCs and individuals with TBI connectivity at the local level, an independent samples *t*-test was used to compare mean weighted network strength between individuals with TBI and HCs during rest and task [see Table 4 (A, B)].

**Table 4:** (A) Positive intra- and internetwork strength; (B) negative intra- and internetwork strength.

	TBI mean (SD)	HC mean (SD)	*T*	Cohen’s *d*	*p*-Value (two-tailed)
**(A) Network strength (positive)**					

Task: DMN-self	207.59 (70.19)	167.25 (30.19)	−2.24	0.75	0.03*

Task: DMN-others	642.35 (189.02)	601.69 (123.43)	−0.70	0.25	0.49

Task: task+-self	182.93 (41.36)	182.27 (40.96)	−0.05	0.02	0.96

Task: task+-others	759.60 (171.09)	718.28 (152.32)	−0.72	0.26	0.48

Rest: DMN-self	193.11 (53.55)	159.25 (41.73)	−1.97	0.71	0.06

Rest: DMN-others	556.50 (195.57)	570.63 (187.37)	0.28	−0.07	0.79

Rest: task+-self	178.73 (26.84)	154.91 (37.49)	−2.13	0.71	0.04*

Rest: task+-others	649.59 (96.39)	613.42 (71.30)	−1.18	0.43	0.24

**(B) Network strength (negative)**					

Task: DMN-self	37.01 (16.90)	0.00 (0.00)	−9.55	3.10	<0.001*

Task: DMN-others	495.48 (132.42)	233.62 (68.31)	−7.38	2.49	<0.001*

Task: task+-self	64.75 (20.50)	0.00 (0.00)	−13.77	4.47	<0.001*

Task: task+-others	480.50 (124.12)	135.92 (43.55)	−11.20	3.70	<0.001*

Rest: DMN-self	34.30 (14.58)	37.25 (15.09)	0.57	−0.20	0.58

Rest: DMN-others	439.02 (84.59)	408.76 (93.19)	−0.97	0.34	0.34

Rest: task+-self	57.35 (22.05)	44.87 (10.66)	−2.15	0.72	0.04*

Rest: task+-others	461.08 (105.50)	392.75 (71.73)	−2.21	0.76	0.03*

*DMN, default mode network; HC, health control; TBI, traumatic brain injury*.

*p < 0.05.

**p < 0.001.

Compared to the HC sample, individuals with TBI showed positive hyperconnectivity *within* the DMN during task and *within* the task-positive network during rest. In other words, ROIs within the network not typically associated with a given state are oscillating together more strongly in TBI. The TBI sample showed enhanced negative connections within the DMN and task-positive network, respectively, and increased between network connectivity (dedifferentiation in terms of negative connections) during task; during rest there were enhanced negative connections within the task-positive network and between the task-positive network and other ROIs.

Taken together, these findings do not support our hypothesis that positive hyperconnectivity would occur through dedifferentiation of networks. In fact, individuals with TBI were hyperconnected within the DMN during task and within the task-positive network during rest. Increased between-network connectivity, which would be suggestive of dedifferentiation were it observed in TBI compared to HCs, did not differ between groups. In terms of negative connectivity, both dedifferentiation though increased between-network connectivity and increased differentiation within networks was observed compared to HCs (with the exception of within the DMN and between the DMN and other ROIs during rest).

### Relationship with Behavior

Prior to testing the relationship between connectivity and behavior, independent samples *t*-tests were implemented in order to observe whether group differences existed on any cognitive measures. No statistical group differences between TBI and HC were observed on any of the five measures examined.

To examine how hyperconnectivity in specific networks relate to behavior, weighted network strengths within networks that showed differences between TBI and HCs were correlated with performance on five neuropsychological tests typically sensitive to deficits commonly reported in TBI. Positive hyperconnectivity within the DMN during task was associated with better performance on Digit Span Backward, a measure of working memory [*R*^2^(18) = 0.28, *p* = 0.02]. In other words, increased dedifferentiation of networks during task was associated with better working memory. Positive hyperconnectivity within the task-positive network during rest was not associated with behavior. Among networks that differed in terms of negative connectivity between TBI and HCs (see Table 5), negative hyperconnectivity between the DMN and ROIs outside of the DMN during rest corresponded with better (faster) performance on the VSAT, a measure of processing speed [*R*^2^(19) = −0.229, *p* = 0.045].

**Table 5:** Relationship between cost and behavior among hubs.

Hub	Neuropsychological measure			
	Trails A^a^	Trails B^a^	Digit span forward	Digit span backward
**Task**				
Middle frontal gyrus	*r* = 0.554*; *p* = 0.017	*r* = 0.613*; *p* = 0.007	*r* = −0.147; *p* = 0.562	*r* = 0.163; *p* = 0.517
Middle temporal gyrus	*r* = 0.377; *p* = 0.123	*r* = 0.484*; *p* = 0.041	*r* = 0.037; *p* = 0.883	*r* = 0.383; *p* = 0.117
Angular gyrus	*r* = 0.396; *p* = 0.104	*r* = 0.455; *p* = 0.058	*r* = 0.078; *p* = 0.760	*r* = 0.040; *p* = 0.875
**Rest**				
Middle frontal gyrus	*r* = 0.471*; *p* = 0.048	*r* = 0.578*; *p* = 0.012	*r* = −0.064; *p* = 0.800	*r* = 0.289; *p* = 0.245
Middle temporal gyrus	*r* = 0.377; *p* = 0.123	*r* = 0.485*; *p* = 0.041	*r* = 0.037; *p* = 0.883	*r* = 0.383; *p* = 0.117
Angular gyrus	*r* = 0.396; *p* = 0.104	*r* = 0.455; *p* = 0.058	*r* = 0.078; *p* = 0.760	*r* = 0.040; *p* = 0.875

*^a^Higher scores are indicative of worse performance; therefore, positive correlations indicate a negative relationship between cost and behavior. No correlations survive statistical corrections. Data are interpreted based upon effect size and consistency of findings*.

*p < 0.05.

### Hubs Driving Network Changes and Their Relationship with Behavior

**Figure 4:**
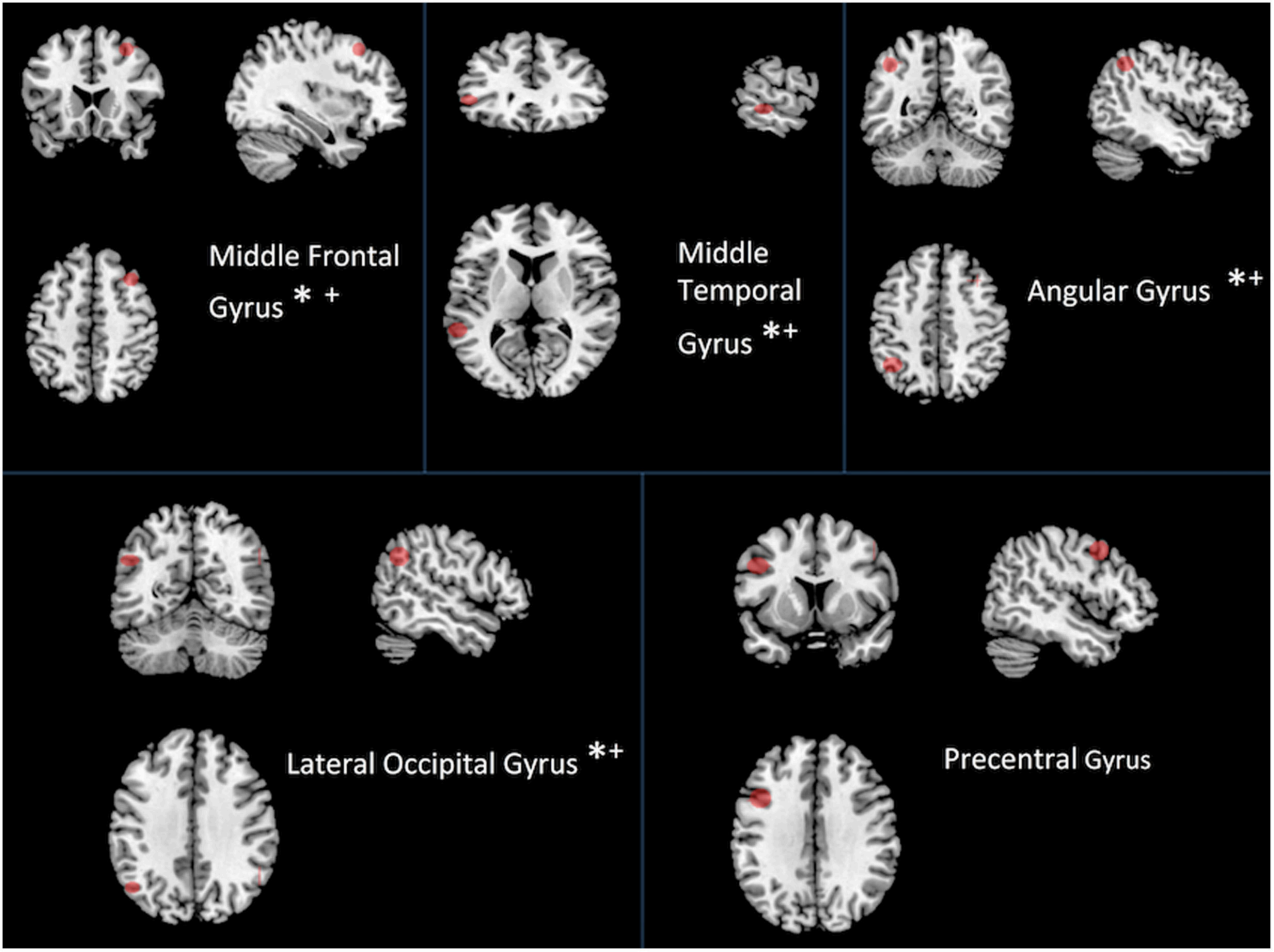
Most costly hubs during both rest and task. Asterisk indicates correlation with behavior (see Table 5). An asterisk (*) indicates a relationship between cost during task and performance; a plus sign (+) indicates a relationship between cost during rest and performance.

We examined the most costly nodes, defined as the sum of the products of Euclidean distance of positive significant edges and their correlation strength, to determine the hubs accounting for changes at the network level within individuals with TBI. Costly nodes were defined as nodes that were greater than 1.5 SDs above the grand mean in terms of cost during both rest and task, respectively. Interestingly, results revealed that the mostly costly hubs were the same five during both rest and task (see Figure 4). Hubs identified *a priori* were then correlated with performance on four neuropsychological tests (see Table 5). To limit the number of comparisons made, the relationship between cost of two hubs related to sensory processing, the precentral gyrus and the lateral occipital gyrus, and behavior was not tested.

Of the five most costly hubs, cost within the middle frontal gyrus and middle temporal gyrus was correlated with worse behavior during both rest and task, while greater cost within the angular gyrus and the lateral occipital gyrus during task was marginally associated with worse performance (see Table 5 for correlations and *p*-values). Though one of the most costly hubs during both rest and task, the precentral gyrus cost did not correspond with behavioral performance.

## Discussion

Neural networks are thought to be structured in a way that maximizes efficiency while minimizing cost (2, 9, 11), and differentiation of networks during different cognitive states could be reflective of such organization (43). The present study demonstrated hyperconnectivity at the whole brain level in individuals with TBI compared to HCs, with significant differences in connectivity at the network level. Connectivity in the DMN during task was found to be a predictor of cognitive performance. Overall, network analyses do not support dedifferentiation of networks in individuals with TBI. Rather, these data indicate that increased connectivity was most evident, possibly counterintuitively, within the DMN during task and within the task-positive network during rest.

One of the primary differences between groups with regard to network response was in measurement of negative functional connectivity. Results of negative connectivity analyses somewhat mirrored positive connectivity in that during rest, the task-positive network showed hyperconnectivity, as did the DMN during task. However, in addition, the task-positive network was negatively hyperconnected during task. Furthermore, there was support for increased differentiation for negative connectivity; within network hyperconnectivity was noted between the task-positive network and other ROIs during both rest and task and between the DMN and other ROIs during task.

In general, brain injury can result in enhanced connectivity of large-scale network hubs (44). However, contrary to our hypothesis that hyperconnectivity would occur through dedifferentiation of networks, our results reveal stronger intranetwork positive connectivity in the TBI sample. This finding was perhaps counterintuitively observed in the DMN during task and the task-positive network during rest. Though our results did not support our hypothesis, they are congruent with results reported in a recent study by Sours et al. (45), in which individuals with mTBI showed increased functional connectivity within the DMN, but not task-positive network during a two-back task. Interestingly, this group also found decreased segregation between task-positive networks and the DMN, which could be interpreted as dedifferentiation of networks with increased cognitive load. The one-back task has relatively low cognitive load, and it is possible that we would have observed dedifferentiation with greater cognitive load. In the current study, negative hyperconnectivity occurred through both increased within-network connectivity and between-network connectivity, potentially indicative of enhanced differentiation. This is inconsistent with findings reported by other groups who have examined negative functional connectivity in TBI. For example, Kasahara et al. (46) found that individuals with TBI showed reduced negative connectivity between motor regions that were negatively connected in HCs, suggesting reduced dissociation of these networks. As the authors note, because they did not examine these differences in the context of behavior (e.g., frequency of errors in motor tasks), it is difficult to determine the relationship of the lack of negative connectivity with behavior. Sours et al. (39) observed negative connectivity in mTBI between the DMN and bilateral insular cortex, left premotor, and bilateral supramarginal gyrus in HCs, but not individuals with mTBI during resting state. These results would be suggestive of dedifferentiation of networks since the networks would be less segregated, yet our results only partially support this finding.

Positive hyperconnectivity within the DMN during task was associated with better working memory, but similar effects in the task-positive network during rest showed no relationship with cognition. Greater between-network negative connectivity between the DMN and other ROIs during rest was associated with faster processing speed within individuals with TBI. Other measures of negative connectivity did not show a relationship with behavior. In the mTBI literature, negative connectivity was associated with measures of episodic memory (39), which were not included in this study.

The most costly hubs among individuals with TBI were the same during rest and task. Cost of four of the five hubs each showed a strong negative relationship with a measure of set-shifting, an area of cognition often impaired following injury (see Figure 4 and Table 5). Cost within the middle frontal gyrus was associated with worse performance on a measure of psychomotor speed. No relationship was observed between hub cost and measures of attention and working memory. That increased connectivity within the DMN during task was associated with *better* cognitive functioning, while increased cost was associated with *worse* cognition is somewhat surprising. The apparent discrepancy may be due to network cost being more strongly related to different cognitive domains (i.e., connectivity more to the domain of working memory, whereas cost is more sensitive to psychomotor speed and set-shifting, involving a component of speed that might be more strongly influenced by Euclidean distance).

The findings presented above are consistent with the broader literature, where hyperconnectivity is a response to neural disruption (36, 37), and specifically with the TBI literature, where hyperconnectivity has been observed in humans (15–19, 23) as well as animal models (47) in TBI. However, unlike the aging literature, which shows a pattern of more distributed network representation through dedifferentiation of networks (26–31), our findings show a strengthening of connections within networks. This is consistent with other findings where patterns of enhanced connectivity within specific networks such as the DMN have been positively linked to behavior (6, 16, 45). The current study expands upon this literature by characterizing changes during both rest and task within and between specific networks during both rest and task and establishing relationships with behavior.

### Limitations and Future Directions

While the use of graph theory to explore functional connectivity has been shown to be a sensitive marker postinjury, there is concern that graph theory metrics of functional connectivity are somewhat limited as a specific marker, given the multifactorial mechanisms that result in hyperconnectivity (36). Though this study was limited in terms of sample size, perhaps with greater power, distinct cognitive profiles may be linked to specific functional connectivity responses post injury. It should be noted the study sample was taken from distinct studies, and therefore had different exposures to the MRI environment. Direct examination of the two TBI subgroups (i.e., previous exposure and no previous exposure) revealed nearly identical network findings (e.g., within DMN connectivity during task for TBI participants with previous task exposure, M = 210.12, SD = 91.89, and TBI participants without previous task exposure, M = 206.92, SD = 67.25, did not differ *p* = 0.94). Therefore, we do not anticipate that exposure to the MRI environment accounts for the findings reported here.

The direction of the relationship between connectivity and cognitive functioning remains largely unknown. Though our results suggest that increased connectivity is associated with better performance, we anticipate this relationship is likely nonlinear. Given our results, and as studies continue to elucidate specific relationships between connectivity and cognitive functioning (i.e., establishing within/between which networks, during which state, and specific hubs), future directions might consider exploring whether manipulating functional connectivity impacts cognitive functioning *via* transcranial magnetic stimulation (48, 49).

## Conclusion

Differences in connectivity between TBI and HC are most salient at the network level, which may be most relevant to specific cognitive changes after injury. Results suggest that the relationship between increased connectivity and cognitive functioning depends on which state and within which network the hyperconnectivity occurs, with evidence for positive connectivity increases within the DMN being associated with better performance during task and increased negative between-network connectivity between the DMN and other ROIs associated with better performance during rest.

## Ethics Statement

This study was carried out in accordance with the recommendations of the Pennsylvania State University Institutional Review Board (IRB) with written informed consent from all subjects. All subjects gave written informed consent in accordance with the Declaration of Helsinki. The protocol was approved by the Pennsylvania State University IRB.

## Author Contributions

RB, AR, and FH developed the methodology and designed the experiments and analysis. AR provided conceptual advice on computational aspect of this work. RB, UV, EG, EB, and FH provided conceptual advice on clinical utility of this work. AR and RB conducted the computational experiments. RB, UV, EG, EB, and FH collected the traumatic brain injury data. RB and AR preprocessed the fMRI data. RB, UV, EG, EB, and FH wrote the manuscript.

## Conflict of Interest Statement

The authors declare that the research was conducted in the absence of any commercial or financial relationships that could be construed as a potential conflict of interest.
