# Corrigendum: Dedifferentiation Does Not Account for Hyperconnectivity after Traumatic Brain Injury

**DOI:** 10.3389/fneur.2017.00674

**Published:** 2017-12-13

**Authors:** Rachel Anne Bernier, Arnab Roy, Umesh Meyyappan Venkatesan, Emily C. Grossner, Einat K. Brenner, Frank Gerard Hillary

**Affiliations:** ^1^Department of Psychology, Pennsylvania State University, University Park, PA, United States; ^2^Social Life and Engineering Sciences Imaging Center, University Park, PA, United States; ^3^Department of Neurology, Hershey Medical Center, Hershey, PA, United States

**Keywords:** traumatic brain injury, TBI, functional connectivity, dedifferentiation, graph theory

In the original article, there was an error. The Conclusion of the Abstract incorrectly stated, “The primary hypothesis that hyperconnectivity occurs through increased segregation of networks, rather than dedifferentiation, was not supported.”

A correction has been made to the Abstract, Conclusion. It now correctly states, “The primary hypothesis that hyperconnectivity occurs through dedifferentiation was not supported.”

The authors apologize for this error and state that this does not change the scientific conclusions of the article in any way.

The original article was updated.

## Conflict of Interest Statement

The authors declare that the research was conducted in the absence of any commercial or financial relationships that could be construed as a potential conflict of interest.

